# Avoiding stress on non-lexical material in nouns and verbs: predictable verb prosody in Serbo-Croatian stress standard varieties

**DOI:** 10.1515/ling-2020-0111

**Published:** 2023-08-11

**Authors:** Marko Simonović, René Kager

**Affiliations:** Institute of Slavic Studies, University of Graz, Graz, Austria; Institute of Linguistics, Utrecht University, Utrecht, Netherlands

**Keywords:** lexical categories, Optimality Theory, Serbo-Croatian, stress assignment

## Abstract

We consider two asymmetries reported in the literature on word prosodic systems: the tendency to allow more prosodic contrast in nouns than in verbs and the tendency to avoid stress on functional material. We focus on the interaction between these two tendencies and propose a formal mechanism to handle this interaction couched in Optimality Theory. In a case study on a group of standard Serbo-Croatian varieties that have predictable stress in verbs but contrastive stress in nouns, we develop an analysis of predictable and morphologically conditioned stress assignment. Our analysis features a family of constraints militating against stress on non-lexical material on three different levels: stress on inflection proper (*Infl-Stress-1), stress on non-lexical material in the locality domain of inflection (*Infl-Stress-2), and stress on non-lexical material altogether (*Infl-Stress-3). Analyzing a class of prosodically exceptional denominal and borrowed verbs, we show that lexical-category effects exist between and within categories: denominal verbs allow exceptional preservation of nominal stress, which leads to additional prosodic contrast in this class of verbs. Finally, we explore the option of subsuming exceptionally contrast-preserving borrowed verbs under denominal verbs, offering arguments in favor of the hotly debated view from the literature that verbs are universally borrowed as denominal*.*

## 1 Introduction

Word prosodic systems in which prosody interacts with the morphological structure are amply attested crosslinguistically. Among the few (nearly) universal tendencies in this interaction are the following two:

a. Nouns allow more prosodic contrast than verbs (see Smith 2011 for an overview) and
b. Lexical morphemes are more prone to receiving prosodic prominence than functional morphemes (Doner 2017; Revithiadou 1999).

Both of these tendencies show that at least some morphosyntactic structure is visible to prosody. As such, these tendencies are important windows into the architecture of grammar. In the literature so far, especially in the accounts couched in Optimality Theory, several mechanisms have been introduced to handle (a) and (b).

Regarding (a) Smith (2011) found an asymmetrical typological pattern such that virtually all languages with phonological differences between verbs and nouns allow more phonological contrast in nouns and, furthermore, that such differences remain restricted to prosody. The asymmetry between verbs and nouns has led phonologists to propose category-specific constraints, such as Noun Faithfulness (see Smith 2001 for a discussion), which specifically protects lexical contrast (e.g., lexical stress, tone) in nouns.

Regarding (b) Revithiadou (1999) proposes the constraint **HeadStress** , a markedness constraint that militates against unstressed morphological heads, defined as “elements that assign a syntactic label to the word and determine its class and gender” (Revithiadou 1999: 1). Since Revithiadou assumes that inflectional material is never head material, stems will naturally have a higher propensity to be stressed than inflectional material. Simonović and Kager (2020) use a derivative of Revithiadou’s constraint, called **StemStress** to account for several diachronic changes in standard Serbo-Croatian.

What is prominently lacking from the literature so far, are analyses of the interaction between a) and b). In order to start filling this gap, we consider a group of varieties of standard Serbo-Croatian in which verbs, unlike nouns, have predictable and inflection-avoiding stress. Moreover, we identify a series of systematic exceptions to the verbal stress pattern and argue that these can be handled by assuming exceptionally contrast-preserving nominal structure.

Our analysis aims to present an explicit toolbox for analyzing interactions between lexical categories and morphosyntactic structure in prosodic systems sensitive to this type of information.

The rest of this article is organized as follows. In Section 2 we introduce the varieties on which we focus here: the standard stress varieties of Serbo-Croatian. Section 3 contains the core of our article. First, we provide the necessary background information on Serbo-Croatian verb morphology. We then introduce our data set and finally present our OT analysis of the verbal domain. In Section 4 we turn to a set of exceptional verbs, which we analyze as enjoying special protection due to the fact that they incorporate nominal structure. Section 5 concludes the article.

## 2 Stress standard Serbo-Croatian and its verbal system

**Figure 1:**
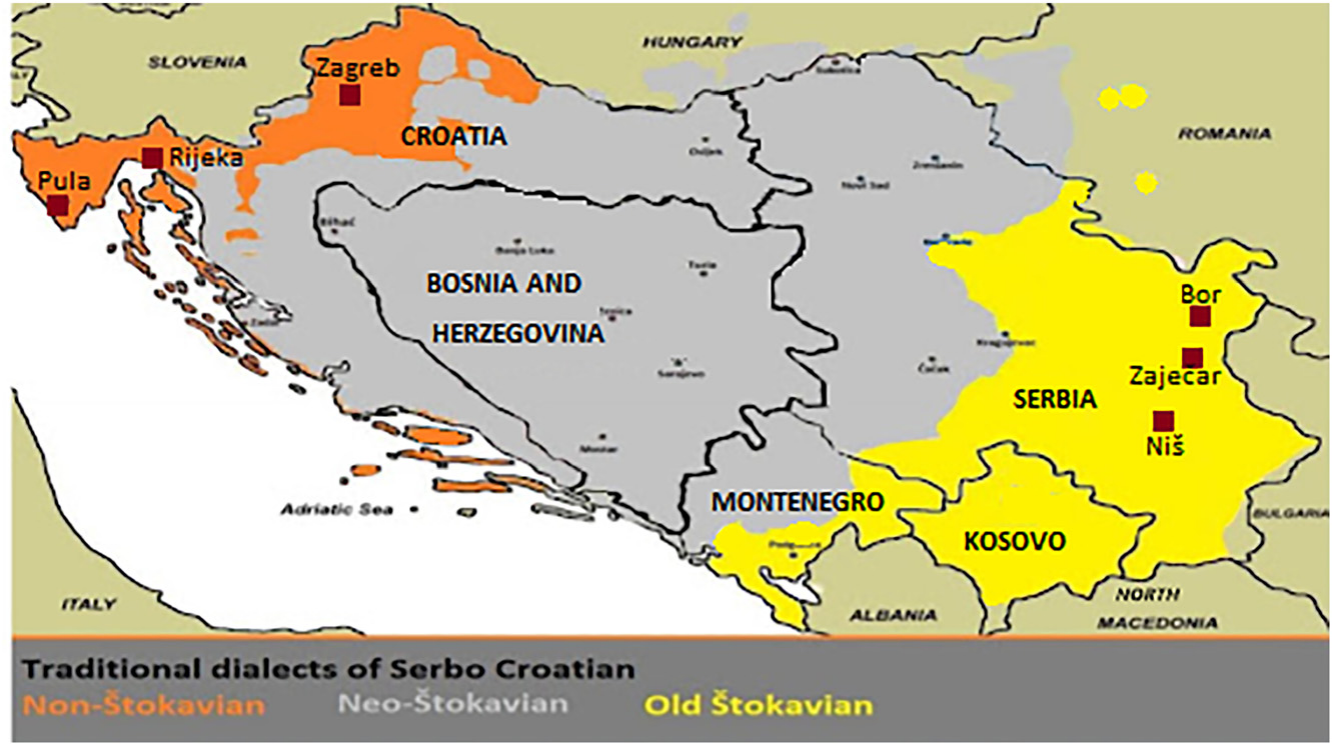
Traditional dialects of Serbo-Croatian.

Standard Serbo-Croatian has two groups of varieties that differ in prosody. On the one hand, there are pitch-accent varieties with contrastive vowel length, spoken in what is traditionally considered the Neo-Štokavian dialect area (e.g., in Osijek, Zadar, Split, Novi Sad and Belgrade). On the other hand, there are stress varieties without contrastive vowel length, spoken in cities in what are traditionally considered Old-Štokavian and Non-Štokavian dialect areas (e.g., in Zagreb, Rijeka, Pula, Niš, Zaječar and Bor). For instance, while pitch-accent speakers pronounce the word *pogledamo* ‘we look’ as [ˈpoglédaːmo]1We use the IPA representations for stress (ˈta), high tone (tá) and vowel length (ta:). (with stress on the first syllable, high tone on the second, and a long third syllable), stress speakers will pronounce it as [poˈgledamo] (with the stressed second syllable and no contrastive tone or length information). More data are provided in (1) and a somewhat simplified dialect map is shown in Figure 1. The map shows the location of the stress-standard-speaking cities mentioned above, as well as the traditional dialect classification, ignoring some smaller dialect groups (e.g., Slavonian), which have little or no influence on the implementation of the standard prosody in the respective area.

(1)

**Table:** 

a.	Pitch-accent standard	*ˈpogléd-a-ti*	*ˈpogléd-aː-mo*
		look-theme-inf	look-theme-1pl
		‘to look’	‘we look’
		*zaˈbol-é-ti*	*zaˈbol-íː-mo*
		hurt-theme-inf	hurt-theme-1pl
		‘to hurt’	‘we hurt’
b.	Stress standard	*poˈgled-a-ti*	*poˈgled-a-mo*
		look-theme-inf	look-theme-1pl
		‘to look’	‘we look’
		*zaˈbol-e-ti*	*zaˈbol-i-mo*
		hurt-theme-inf	hurt-theme-1pl
		‘to hurt’	‘we hurt’

All the forms in (1) have the structure stem + theme vowel + inflectional ending. The stress placement of the specific forms in (1b), used to illustrate the stress standard varieties, can be shared with various (both Neo-Štokavian and non-Neo-Štokavian) varieties, but there is no other described variety in which the four forms co-occur. For example, stem-final stress of *poˈgled-a-ti* and *poˈgled-a-mo* is attested in many Old Štokavian and Non-Štokavian dialects. However, these dialects stress the other two forms on the theme: *zaboˈl-e-ti* and *zaboˈl-i-mo* or equivalents thereof. In sum, stress placement in stress standard Serbo-Croatian is a unique combination of patterns, not encountered in any traditional dialect and constitutes a standardization-induced innovation.

In stress standard SC the stress position in verbs is predictable, which is again in stark contrast with all other described SC varieties, both stress and pitch-accent ones. As a first approximation, we can say that verbal stress in stress standard SC is typically stem-final. This suggests that stress standard SC verbs have no prosodic information in their lexical representation, unlike verbs in pitch-accent varieties, where verb prosody is distinctive (see the contrast between the verbs in (1)). Noun prosody, on the other hand, is contrastive in all varieties, as illustrated by the words in (2), which only differ in stress placement.

(2)

**Table:** 

a.	Pitch-accent standard	*ˈnóvine*	*noˈviné*
b.	Stress standard	*ˈnovine*	*noˈvine*
		‘newspapers’	‘novelties’

In sum, pitch-accent standard SC has lexical prosody in both nouns and verbs, whereas stress standard SC only has lexical prosody in nouns, but not in verbs. As such, stress standard SC is similar to Spanish (Roca 2005) and Hebrew (Becker 2003; Bolozky 2000). However, both of these languages have prosodic differences in verbal inflectional affixes, some being lexically stressed, while others are stressless. For example, Spanish has minimal pairs of the type *ám-o* ‘I love’ versus *am-ó* ‘s/he loved’. Stress standard SC has no such pairs, as all verbal affixes are lexically unstressed. Moreover, both Spanish and Hebrew are described as having trisyllabic-window effects, which is a confounding factor in an analysis of the lexical prosody of their verbs, potentially obscuring any stem-stress effects.

A comprehensive overview of stress standard SC stress placement (in Sections 3 and 4) shows that there are several classes of apparent as well as genuine exceptions to stem-final stress. In our analysis couched in Optimality Theory, we show that all stress standard SC verbs follow the same grammar. The driving force is a family of morpho-prosodic constraints to which Simonović and Kager (2020) refer as **StemStress** , defining it as “a markedness constraint that requires prosodic prominence on the stem, regardless of whether it is lexically sponsored or not” (Simonović and Kager 2020: 307). As our overview will show, while for canonical verbs (which constitute a huge majority of verbs) a single constraint, **StemStress** is sufficient and its implementation is straightforward (the stem domain being whatever precedes the theme vowel), more complex environments reveal more fine-grained preferences, as well as less straightforwardly parsable structures. For example, when the stem contains no stressable material, the choice between stressing a theme vowel and stressing a negation particle cannot be resolved by **StemStress** . Yet rather than displaying free variation, our data show a systematic preference for the latter stress placement. The unpacking of the constraint **StemStress** will lead us to formulate a family of negative constraints termed *Infl-Stress , which all militate against stress on non-lexical material.

Before turning to the analysis of the specific cases, we present the general structure of the Serbo-Croatian verb in the following section.

## 3 Stress standard Serbo-Croatian and its verbal system

### 3.1 Serbo-Croatian verb morphology

Serbo-Croatian verb forms minimally have the structure stem + theme vowel + inflectional ending(s). In (3) we give examples of verb forms represented by the infinitive and the first-person plural present-tense form.

(3)

**Table:** 

Verbal forms in Serbo-Croatian (stem-theme-inflection)		
a.	*pit-a-ti*	‘to ask’
	*pit-a-mo*	‘we ask’
b.	*plov-i-ti*	‘to sail’
	*plov-i-mo*	‘we sail’
c.	*zasp-a-ti*	‘to fall asleep’
	*zasp-i-mo*	‘we fall asleep’
d.	*kre-nu-ti*	‘to leave’
	*kre-ne-mo*	‘we leave’
e.	*pas-∅-ti*	‘to graze’
	*pas-e-mo*	‘we graze’

As can be seen from the examples above, some verbs take the same theme vowel in all forms (*pit-a-ti* and *plov-i-ti*), while others take different theme vowels in different forms (all the other verbs in (3)). The theme vowels are themselves not an expression of an inflectional head but their selection is nonetheless influenced by the adjacent inflection. For most varieties, the distribution of the two theme vowels in the verbal paradigm can be analyzed as related to finiteness. If the adjacent inflection is non-finite, the theme vowel illustrated in the infinitive forms above will show up (e.g., the theme vowel *a* in *zasp-a-ti,* and *nu* in *kre-nu-ti* above), otherwise the alternate theme vowel will be inserted (e.g., the theme vowel *i* in *zasp-i-mo,* and *ne* in *kre-ne-mo*). Because the two exponents of the theme vowel are unpredictable based on each other, we illustrate Serbo-Croatian verbs using a non-finite and a finite form: the infinitive (ending: *-ti*) and the first-person-plural present-tense form (ending: *-mo*). We will also include the negated version of the present-tense form in cases where there is a prosodic interaction with the negative particle *ne* (e.g., in *né d-a-mo* ‘we don’t give’).

Theme vowel combinations are the most common way of describing Serbo-Croatian conjugation classes (see, e.g., Jelaska and Bošnjak-Botica [2019] for a recent overview). However, as we will also show in the following section, providing a complete list of theme-vowel classes is not at all straightforward. While an overwhelming majority of verbs can easily be classified into theme-vowel classes, there are many smaller classes that lend themselves to different possible analyses.

For easier presentation, until we refine the structure, we will reserve the term ‘stem’ for whatever material precedes the theme vowel, including prefix material (but not the negation particle), while everything added after the theme vowel will be termed ‘inflection’. Throughout the article, we will use the term theme vowel, although the theme can also contain a consonant (e.g., in *kre-nu-ti*) or be empty (e.g., in *pas-∅-ti*).

### 3.2 Dataset

Stress standard Serbo-Croatian is the de facto implementation of standard Serbo-Croatian in the urban centers outside of the traditional Neo-Štokavian area in both Serbia and Croatia. Despite its being extremely widespread, there are no detailed descriptions of this system. The modest literature consists of remarks on Zagreb SC in Kapović (2018) and general remarks in Simonović and Kager (2017, 2020. While the differences between the Croatian and the Serbian stress standard varieties are relatively few, in this article, we focus on the Serbian varieties.

In order to obtain a good overview of the stress standard SC situation, we assembled a database of the 3,000 most frequent verbs in the Serbian variety of standard Serbo-Croatian. For this purpose, we used the web corpus srWac (Ljubešić and Klubička 2014). We asked four stress standard SC speakers (two from Niš, one from Bor and one from Zaječar) to pronounce one finite and one non-finite form of each verb. The speakers were asked to use the variety they would speak when talking to a stranger from their own hometown in a relatively formal setting (e.g., at a doctor’s or a government office).2The dataset can be accessed at: https://doi.org/10.5281/zenodo.6606901.


The 3,000 verbs were classified based on the theme-vowel combination. As foreshadowed in the previous section, there is a dichotomy, which will be crucial for our analysis in Section 3.3, between verbs in which theme vowels can be unproblematically isolated and those in which different analyses are a priori possible.

In order to illustrate approximate patterns of distribution, we provide a quantitative overview of the encountered types for the transparent group (Table 1) and the nontransparent group (Table 2). The transparent types are named after their respective theme-vowel combinations. The non-transparent types are illustrated using an example verb.

**Table 1:** The transparent verb types.

Type (TV combination)	i/i	a/a	a/je	nu/ne	a/e
Example	*plov-i-ti* *plov-i-mo* ‘sail’	*pit-a-ti* *pit-a-mo* ‘ask’	*pis-a-ti* *piš-e-mo* ‘write’	*kre-nu-ti* *kre-ne-mo* ‘leave’	*greb-a-ti* *greb-e-mo* ‘scratch
Number (% of dataset)	966 (32.20)	929 (31)	181 (6)	105 (3.5)	78 (2.6)
Type (TV combination)	e/i	a/i	e/e	∅/e	
Example	*žel-e-ti* *žel-i-mo* ‘wish’	*zasp-a-ti* *zasp-i-mo* ‘fall asleep’	*um-e-ti* *um-e-mo* ‘be able	*tres-∅-ti* *tres-e-mo* ‘shake’	
Number (% of dataset)	65 (2.2)	37 (1.2)	36 (1.2)	17 (0.6)	

**Table 2:** The nontransparent verb types.

Type	*ispitivati* *ispitujemo* ‘examine’	*školovati* *školujemo* ‘school’	*povući* *povučemo* ‘pull’	*dovesti* *dovedemo* ‘bring’	*popiti* *popijemo* ‘drink up’
Number (%)	179 (6)	169 (5.6)	96 (3.3)	50 (1.7)	44 (1.5)
Type	*ustati* *ustanemo* ‘get up’	*prodavati* *prodajemo* ‘sell’	*doneti* *donesemo* ‘bring’	*obuti* *obujemo* ‘put on’	*pljuvati* *pljujemo* ‘spit’
Number (%)	17 (0.6)	13 (0.4)	13 (0.4)	4 (0.1)	1 (0.0)

The transparent types constitute 80 % of the verbs in our data set. Within this group, the two largest classes (a/a and i/i) are far more frequent than all others and account for more than 63 % of all verbs in the language. The only class that always involves phonological alternation is a/je, where the theme *je* palatalizes the preceding consonant, so e.g., /pis-je-mo/ comes out as [piʃemo]. The existence of both a/je and a/e as separate theme vowel classes means that some verbs will be ambiguous between these two classes. For instance, the verb *opirati∼opiremo* ‘resist’, can be analyzed as belonging to either the a/je or the a/e class, because underlying /rj/ yields [r]. In such cases, we non-crucially assigned the verbs in question to the a/e class.

Nontransparent classes account for 20 % of all verbs and allow multiple possible parsings into stems and theme vowels, as also reflected in the different treatments in traditional descriptions, which resulted in different classifications (see, e.g., Jelaska and Bošnjak-Botica 2019 for a recent overview). This ambiguity will be elucidated using the two most common types from Table 2, illustrated by *ispitivati∼ispitujemo* ‘explore’ and *školovati∼školujemo* ‘school’, respectively.

These two types have alternating sequences *iva∼uje* and *ova∼uje*. One possible analysis is subsuming the two types under the transparent type a/je and assuming that affixes *iv∼u* and *ov∼u* display theme-vowel-triggered allomorphy. This would make it plausible to approach other, smaller types in a similar way, e.g., assuming parsings such as *prodav-a-ti∼proda-je-mo* and *pljuv-a-ti∼plju-je-mo.* While this allows grouping a multitude of apparently stray types under a transparent type, it also means assuming a large amount of stored allomorphy, possibly missing some predictable information. For instance, in the four types just discussed, we have to assume that it is purely accidental that the stem allomorphs that appear before the theme vowel *a* end in the consonant *v* in three out of the four cases. The same goes for the vowel *u* preceding the theme *je.*

There is a second a priori available approach, however. In order to avoid massive allomorphy, it is possible to assume that the entire alternating sequences in the nontransparent classes are thematic, so for *ispitivati∼ispitujemo* ‘explore’ and *školovati∼školujemo* ‘school’ the thematic sequences would be iva/uje, ova/uje. While this resolves the issue of allomorphy, it increases the list of theme-vowel classes in Serbo-Croatian and assumes many theme vowels which only exist in one, potentially very small class. For instance, the class va/je would unite 14 verbs from the types *proda-va-ti∼proda-je-mo* and *plju-va-ti∼plju-je-mo*. Moreover, the segmental content of the alternating thematic sequence va/je would, on the one hand, suspiciously contain that of the class a/je and be contained by those of two more numerous thematic classes: iva/uje and ova/uje. As we saw above in the discussion of the transparent classes a/je and a/e, such containment relations make verbs analyzable as belonging to various classes, without a clear criterion to distinguish between different analyses.

Third, an analysis is possible that allows multiple theme vowels per verb form. In this case, the last discussed four classes would be possibly analyzed in terms of *i-va∼u-je* and *o-va∼u-je* containing *va∼je.* A potential problem here would be that the themes that precede *va* and *je* are not elsewhere attested theme vowel combinations (*i∼u* and *o∼u*), which in turn means that we may end up with more theme vowel types than in the previous analysis.

The upshot of this discussion is that the nontransparent verbs present speakers with analytical choices, but mere consideration of the segmental content does not give a clear clue how these choices are made and if they are made by all speakers in the same way. As we will show in Section 3.3.2, our prosodic data testify that the analytical ambiguity has been resolved by the native speakers consistently for each particular type, as indicated by the position of stress. Thereby prosody becomes a litmus test for morphological structure.

One important aspect of the data to keep in mind is that the quantitative relation between the transparent and nontransparent types is 8:2. In 80 % of the verbs there is no doubt as to where the theme vowel begins. Their analysis provides a “blueprint” for resolving the morphological ambiguities of nontransparent verbs. We will start our data analysis in the following subsection by turning to the transparent verbs.

### 3.3 Data and OT analysis

#### 3.3.1 Transparent verbs: StemStress

In this section, we start our analysis by the ‘default of the defaults’: morphologically transparent verbs with stressable stems, i.e., stems containing a vowel. In all verbs with a transparent structure, stress falls on the stem-final syllable, i.e., the syllable preceding the theme vowel. In (4) this is illustrated for the verb *pitati*∼*pitamo* ‘ask’.

(4)

**Table:** 

*ˈpit-a-ti*
‘to ask’
*ˈpit-a-mo*
‘we ask’
*ne ˈpit-a-mo*
‘we don’t ask’

In our OT analysis, the crucial ranking is then **StemStress** >> **Rightmost**. The former constraint (Simonović and Kager 2020) penalizes stress on non-stem material, while the latter (Prince and Smolensky 1993) pulls stress towards the right word edge. The effect of this ranking is stress on the final (only) syllable of the stem, as shown in the tableaux in (5), (6) and (7).

(5)

**Table:** 

/**pit**-a-ti/	**StemStress**	**Rightmost**
☞ a.ˈpitati		**
b. piˈtati	*!	*
c. pitaˈti	*!

(6)

**Table:** 

/**pit**-a-mo/	**StemStress**	**Rightmost**
☞ a.ˈpitati		**
b. piˈtamo	*!	*
c. pitaˈmo	*!

(7)

**Table:** 

/ne=**pit**-a-mo/	**StemStress**	**Rightmost**
a. ˈnepitamo	*!	***
☞ b. neˈpitamo		**
c. nepiˈtamo	*!	*
d. nepitaˈmo	*!	

In the three forms above, the only active constraint was **StemStress.** The constraint **Rightmost** starts playing a role in cases where there is more stem material than just one syllable. This is shown using the prefixed version of *pitati∼pitamo, ispitati∼ispitamo* ‘explore’ (8) and a longer verb *računati∼računamo* ‘calculate’ (9). The tableaux for the present-tense forms of these verbs are shown in (10) and (11).

(8)

**Table:** 

a.	*isˈpit-a-ti*
	‘to explore
b*.*	*isˈpit-a-mo*
	‘we explore’
c.	*ne isˈpit-a-mo*
	‘we don’t explore’

(9)

**Table:** 

a.	*raˈčun-a-ti*
	‘to calculate’
b.	*raˈčun-a-mo*
	‘we calculate’
c.	*ne raˈčun-a-mo*
	‘we don’t calculate’

(10)

**Table:** 

/**is+pit**-a-mo/	**StemStress**	**Rightmost**
a. ˈispitamo		***!
☞ b. isˈpitamo		**
c. ispiˈtamo	*!	*
d. ispitaˈmo	*!

(11)

**Table:** 

/**račun**-a-mo/	**StemStress**	**Rightmost**
a. ˈračunamo		***!
☞ b. raˈčunamo		**
c. račuˈnamo	*!	*
d. računaˈmo	*!	

Note that while in the tableaux the inputs for the two last verbs with disyllabic stems are presented as bimorphemic and monomorphemic respectively, prosody makes no difference between them. As will be further discussed in the following section, prefixes systematically behave as part of the stem (at least when it comes to prosody). In the syntactic literature on Slavic, a dichotomy is observed between the so-called lexical (or internal) prefixes, which typically introduce some non-compositional meaning component, and superlexical (or external) prefixes, which compositionally introduce a quantificational meaning component (see Arsenijević 2006 and references therein). The prefix *iz-* in *ispitati* ‘explore’ is a lexical prefix, as is the prefix *iz-* in the prefixed version of *računati, izračunati* ‘compute’. The stress pattern of the latter verb is, expectedly, *izra*ˈ*čunati*.

Superlexical prefixes only combine with imperfectives, so in order to add a superlexical prefix to the perfective verb *izra*ˈ*čunati*, we need to take an intermediate derivational step and make *izra*ˈ*čunati* imperfective. The imperfective version of *izra*ˈ*čunati* is *izraču*ˈ*navati.* The suffix *av* is one of the ‘secondary imperfectivizers’ in Serbo-Croatian and itself counts as part of the stem, as do all derivational affixes. Now we can finally add the external prefix *na* and obtain *naizračuˈnavati se* ‘have one’s fill of computing’. The latter verb has the expected prosodic pattern, shared with i*zraču*ˈ*navati* and the other 149 verbs ending in this secondary imperfectivizer in our dataset.

From the perspective of prosody assignment, all the verbs discussed here are ‘regular’ in the sense that they have a clearly identifiable theme vowel preceded by stressable material. As we will see in the following section, this stressable stem material can consist only of a lexical prefix. In the next section, we turn to the few verbs which have consonantal stems.

#### 3.3.2 Consonantal stems

The need for unpacking **StemStress** into a family of constraints arises in verbs with consonantal stems, which contain no stressable material. Two such verbs are illustrated in (12) and (13).

(12)

**Table:** 

a.	*ˈzn-a-ti*
	‘to know’
b.	*ˈzn-a-mo*
	‘we know’
c.	*ne zn-a-mo*
	‘we don’t know’

(13)

**Table:** 

a.	*ˈsm-e-ti*
	‘to dare’
b.	*ˈsm-e-mo*
	‘we dare’
c.	*ˈne sm-e-mo*
	‘we don’t dare’

Note that none of these forms is predicted by our current ranking **StemStress** >> **Rightmost**, which predicts final stress in all the forms since **StemStress** does not differentiate between theme vowels and inflectional endings as hosts for stress (both incur violations).

**StemStress** cannot be satisfied without adding stressable material (which is not an option in Serbo-Croatian, due to the high ranking of the anti-insertion constraint **Dep-IO** ) or leaving the word stressless (which is not an option, due to high-ranked **Culminativity** ). So, in our current ranking, **StemStress** passes the decision on to **Rightmost**, which in turn picks the candidate with final stress. However, what we see in the data in (12) and (13) is a system that clearly prefers stressing the theme vowel to stressing agreement morphology, e.g., [*ˈ*znamo]>>[zna*ˈ*mo], and stressing the negative particle to stressing the theme vowel, e.g., [*ˈ*neznamo]>>[ne*ˈ*znamo].

Stressing inflectional morphology (illustrated by *-mo* and *-ti* in our examples) is avoided under all circumstances in stress standard SC and the relevant constraint is therefore undominated. We can formulate this constraint as follows.

**Table:** 

*Infl-Stress-1:	No stress on inflectional material (Tense, Agreement etc.)

The next least desirable structure, which nevertheless surfaces in certain contexts, is stress on theme vowels. As we have seen, in Serbo-Croatian, the exponents of theme vowels are conditioned by the finiteness of Tense (recall the existence of verbs such as *zasp-a-ti* ‘to fall asleep’, *zasp-i-mo* ‘we fall asleep’). Their conditioning by Tense, a piece of inflectional morphology par excellence, makes them undesirable hosts of prosodic prominence, presumably because the theme vowel can be viewed as expressing a Tense feature indirectly. This constraint is formalized below.

**Table:** 

*Infl-Stress-2:	No stress on non-lexical material in the locality domain of inflectional heads.

Now we can turn to the analysis of the verb forms from (12) in the tableaux in (14), (15) and (16). From this point on the ‘unpacked’ ranking *Infl-Stress-1 **>>** *Infl-Stress-2 replaces the constraint **StemStress.** In all the cases already analyzed the two versions will have the same effect: they will enforce stress on the rightmost syllable which does not belong to inflectional morphology or to theme vowels. Note, however, that using the constraints which explicitly state which structures are avoided by stress allows us to circumvent the admittedly ambiguous term stem.

(14)

**Table:** 

/**zn**-a-ti/	*Infl-Stress-1	*Infl-Stress-2	**Rightmost**
☞ a. ˈznati		*	*
b. znaˈti	*!	*

(15)

**Table:** 

/**zn**-a-mo/	*Infl-Stress-1	*Infl-Stress-2	**Rightmost**
☞ a. ˈznamo		*	*
b. znaˈmo	*!	*

(16)

**Table:** 

/ne=**zn**-a-mo/	*Infl-Stress-1	*Infl-Stress-2	**Rightmost**
☞ a.ˈneznamo			**
b. neˈznamo		*!	*
c. neznaˈmo	*!	*	

As previewed in the previous section, prefix material behaves as any other non-inflectional material. As predicted by the current ranking, as soon as a prefix adds stressable material, this added material gets stressed. This is illustrated by the paradigm of the verb *priznati∼priznamo* ‘admit’ in (17).

(17)

**Table:** 

*ˈprizn-a-ti*
‘to admit’
*ˈprizn-a-mo*
‘we admit’
*ne ˈprizn-a-mo*
‘we don’t admit’

The relevant tableaux are shown in (18) and (19).

(18)

**Table:** 

/**pri**-zn-a-mo/	*Infl-Stress-1	*Infl-Stress-2	**Rightmost**
☞ a. ˈpriznamo			**
b. priˈznamo		*!	*
c. priznaˈmo	*!	*

(19)

**Table:** 

/ne=**pri-zn**-a-mo/	*Infl-Stress-1	*Infl-Stress-2	**Rightmost**
a. ˈne priznamo			***!
☞ b. neˈpriznamo			**
c. ne priˈznamo		*!	*
d. ne priznaˈmo	*!	*	

Note that a prefixed verb like *priznati∼priznamo* ‘admit’ behaves identically to the unprefixed monosyllabic *pitati∼pitamo* ‘ask’ in (4). Once again, prosody shows no indication of distinguishing prefixes from (other) lexical material. Also, the prefixed version of *priznati∼priznamo, otpriznati∼otpriznamo* ‘unrecognize’, predictably gets stressed as *ot*ˈ*priznati∼ot*ˈ*priznamo*. As stated in the previous section, while we show the inputs of prefixed verbs in our tableaux as consisting of several pieces, prosody offers no evidence of distinguishing between these two pieces.

This concludes our analysis of transparent verbs, which has established the basic interaction of verb morphology and prosody. This analysis provides a “probe” for resolving the morphological ambiguities of nontransparent verbs, to which we turn in the next section.

Before moving on, a brief comment is in order regarding the negation particle *ne*. The current constraint ranking (with *Infl-Stress-1 and *Infl-Stress-2 replacing **StemStress** ) essentially treats *ne* on a par with lexical material both when it comes to its not being inflectional (or in the locality domain of inflection) and its belonging to the same prosodic word as the verb. Indeed, the prosody of the transparent types does not justify distinguishing between the negation particle and lexical material. Note that this yielded the correct result in Tableau (19) due to **Rightmost** . However, there are prosodic reasons to consider *ne* as non-lexical material.

The prosodic status of *ne* has been addressed in the literature on Serbo-Croatian (see Werle 2009 for an overview and an analysis). Based on Neo-Štokavian facts, Werle argues that *ne* is an *internal proclitic*, making it less word-internal than prefixes but more word-internal than prepositions. Interestingly, despite major prosodic differences, we draw the same conclusion about *ne* in stress standard Serbo-Croatian. In the following section, we will present arguments that the negation particle is distinguished from prefixes in that it is less prone to carrying stress. Furthermore, while *ne* can get stress in the few cases where otherwise stress would end up on pieces of agreement morphology, no preposition ever gets stressed in stress SC (outside lexicalized PPs). Even in the very few nouns which have consonantal stems, in PPs case endings get stressed rather than prepositions, e.g., in *o ˈps-u* ‘about a dog’, *na ˈdn-u* ‘at the bottom’, where the locative case ending *-u* gets stressed*.*

### 3.4 Nontransparent verbs

As argued above, nontransparent verbs are analytically ambiguous and just based on segmental content, it is not clear which analysis is chosen by the speakers, or even if a specific analysis is chosen. However, a system in which prosody is guided by morphological boundaries necessitates analytical choices. The stress placement in the nontransparent verbs indicates that such choices have been made by native speakers, resolving the ambiguities concerning theme vowels. As our occasional references to Croatian stress standard varieties will show, this is the domain where Serbian and Croatian varieties occasionally made different choices, confirming that both options were indeed available and that picking one was a matter of conventionalization.

While for transparent verbs the segmentation into morphological domains was unambiguous and prosody depended on these boundaries, in considering nontransparent verbs, we will use prosody as a clue to establish which of the several possible segmentations was chosen.

In Table 3 we repeat the nontransparent types with stress markings in Serbian stress standard SC.

**Table 3:** The nontransparent verb types with stress marks for *Niš*.

Type	*ispiˈtivati* *iˈspitujemo* ‘test’	*ˈškolovati* *ˈškolujemo* ‘school’	*poˈvući* *poˈvučemo* ‘pull’	*ˈdovesti* *doˈvedemo* ‘bring’	*ˈpopiti* *ˈpopijemo* ‘drink up’
Number (%)	179 (6)	169 (5.6)	96 (3.3)	51 (1.7)	44 (1.5)
Type	*ˈustati* *uˈstanemo* ‘get up’	*proˈdavati* *ˈprodajemo* ‘sell’	*ˈdoneti* *doˈnesemo* ‘deliver’	*ˈobuti* *ˈobujemo* ‘put on’	*ˈpljuvati* *ˈpljujemo* ‘spit’
Number (%)	17 (0.6)	13 (0.4)	13 (0.4)	4 (0.1)	1 (0.0)

The only generalization that can be immediately read from the data in Table 3 is that, just as in transparent verbs, stress never ends up on inflectional endings. For the rest, in the seemingly chaotic picture, there are several strong generalizations.

1. In all infinitive forms which end in *-ti*, the vowel preceding this ending is unstressed.
2. In the small non-transparent group in which the infinitive form ends in *-ći,* the vowel preceding *-ći* is stressed.
3. In all present-tense forms (which end in *-mo* in the examples) the vowel preceding the inflectional ending is unstressed.

Focusing on the infinitive forms for a moment, we can say that the only non-transparent infinitives which allow stress on the syllable directly preceding the ending are the exceptional *ći*-infinitives (3 % of all verbs in our dataset), whereas the common *ti*-infinitives always have the vowel preceding the ending unstressed.

*Ći*-verbs are generally assumed to belong to the type ø/e (which has only 16 transparent members in our dataset, e.g., istrés-ø-ti ∼ istrés-e-mo ‘shake off’). If this is the case, the correct parsing is *povu-ø-ći (*or *povuć-ø-i,* see below*)∼povuč-e-mo*. In most traditional approaches, the ending *-ći* [t͡ɕi] is assumed to be the result of a historical process that fused one velar consonant of the stem and the consonant of the infinitive ending *-ti*, e.g., *povući* then originates from *povukti* (see the discussion in Stevanović 1970, 341–342)*.* In the present-tense form, the stem-final velar got palatalized so that *povuk-e-mo* yielded *povuč-e-mo* [poʋut͡ʃemo]. Our prosodic data seem to testify to this zero-theme analysis of *-ći* forms (while, as we will see below, all *ti*-infinitives have an overt theme). Assuming the parsing *povu-ø-ći/povuć-ø-i∼povuč-e-mo* and our current phonological grammar, we indeed get the correct (stem-final) stress placement: *poˈvu-ø-ći∼poˈvuč-e-mo*.

In all other cases, the infinitive affix *-ti* is segmentally unaffected by the stem and the more canonical parsing is implemented: the vowel preceding the ending *-ti* is viewed as a theme vowel. This even happens in the type *ˈdovesti∼doˈvedemo*, which would traditionally be analyzed as featuring a zero theme, but the prosodic facts indicate that the parsing is *ˈdov-e-sti∼doˈved-e-mo.* The same is true of *ˈdon-e-ti∼doˈnes-e-mo* ‘bring’.

This parsing of all vowels preceding *-ti* as theme vowels reveals some theme vowels that segmentally correspond to already encountered theme vowels (e.g., *i* in ˈ*pop-i-ti*) but it also reveals some themes which have not been assumed before (e.g., *u* in ˈ*ob-u-ti*).

The analysis of the infinitive forms appears to have influenced the analysis of the present-tense forms in some types. If a vowel marked as a theme vowel in the infinitive form reappears in the present-tense forms (e.g., in the type *obuti∼obujemo*), the same theme analysis is in some cases extended to the present-tense forms. This leads to certain present-tense forms being analyzed as having two theme vowels. In other words, once *obuti* is analyzed as *ob-u-ti*, it is harder to analyze *u* in *obujemo* as part of the stem. The attested stress pattern ˈ*obuti∼ˈobujemo* points in the direction of an analysis of *u* as thematic in both forms: ˈ*ob-u-ti∼ˈob-u-je-mo*. This parsing straightforwardly accounts for the stress pattern in the types *ˈpop-i-ti∼ˈpop-i-je-mo* and *ˈobuti∼ˈobujemo.*

However, the double-theme analysis does not extend to all the types in which it is a priori possible. For instance, the type *ustati∼ustanemo* seems available for the same analysis: since *a* is a theme in *ustati* (as attested by the stress pattern *ˈust-a-ti*), this analysis of *a* as a theme could extend to the present-tense form *ustanemo.* However, judging by the attested prosodic pattern, the present-tense form has a single theme *uˈsta-ne-mo*, since the double theme analysis would predict *ˈust-a-nemo. This is an important point where a comparison with the Croatian stress standard varieties is instrumental. At least in Zagreb and Rijeka,*ˈustanemo* is the only possible form, perhaps the most prominent systematic difference between the Serbian and Croatian stress standard varieties.

The generalization for the Serbian varieties is that only verb forms with the theme vowel *je* allow a vowel directly preceding it to be segmented as a theme. This segmentation applies in all cases where *je* is directly preceded by a vowel. This generalization suffices to describe the assignment of double theme vowels in the Serbian stress standard and the algorithm does not need to make reference to the infinitive form. This is further confirmed by the types *proˈdav-a-ti∼ˈprod-a-je-mo* and *ispiˈtiv-a-ti∼iˈspit-u-je-mo,* where the theme is not carried over from the infinitive*.*

The parsing algorithm for Serbian stress standard SC verbs is summarized in (20).

(20)

**Table:** 

Parsing algorithm for Serbian stress standard SC verbs
– Infinitives in *-ći* have a zero theme preceding *-ći.*
– Infinitives in *-ti* have a vocalic theme preceding *-ti.*
– Vowels preceding present-tense endings are (part of) themes.
– All vowels directly preceding the theme *je* are themes as well.

In Table 4, we show all the nontransparent types parsed using the algorithm in (20).

**Table 4:** The nontransparent verb types with parsing based on (20).

*ispiˈtiv-a-ti* *iˈspit-u-je-mo* ‘test’	*ˈškolov-a-ti* *ˈškol-u-jemo* ‘school’	*poˈvu-ø-ći* *poˈvuč-e-mo* ‘pull’	*ˈdov-e-sti* *doˈved-e-mo* ‘bring’	*ˈpop-i-ti* *ˈpop-i-je-mo* ‘drink up’
*ˈust-a-ti* *uˈsta-ne-mo* ‘get up’	*proˈdav-a-ti* *ˈprod-a-je-mo* ‘sell’	*ˈdon-e-ti* *doˈnes-e-mo* ‘deliver’	*ˈob-u-ti* *ˈob-u-je-mo* ‘put on’	*ˈpljuv-a-ti* *ˈplj-u-je-mo* ‘spit’

Having the parsing in place, we can now move on to the OT analysis. For all types where a single theme vowel is isolated in all forms (*poˈvu-ø-ći∼poˈvuč-e-mo, ˈdov-e-sti∼doˈved-e-mo, ˈust-a-ti∼uˈsta-ne-mo* and *ˈdon-e-ti∼doˈnes-e-mo*), our current ranking predicts the full paradigm as is.

Our ranking also unproblematically deals with short verbs with multiple themes, e.g., the prefixless version of *ˈpop-i-ti∼ˈpop-i-je-mo: ˈp-i-ti∼ˈp-i-je-mo* ‘drink’*.* The paradigm is shown in (21) and the relevant tableaux in (22)–(24).

(21)

**Table:** 

a.	*ˈp-i-ti*
	‘to drink’
b.	*ˈp-i-je-mo*
	‘we drink’
	*ne ˈp-i-je-mo*
	‘we don’t drink’

(22)

**Table:** 

/**p**-i-ti/	*Infl-Stress-1	*Infl-Stress-2	**Rightmost**
☞ a.ˈpiti		*	*
b. piˈti	*!	*

(23)

**Table:** 

/**p**-i-je-mo/	*Infl-Stress-1	*Infl-Stress-2	**Rightmost**
☞ a.ˈpijemo			**
b. piˈjemo		*!	*
c. pijeˈmo	*!	*	

Note that candidate a. in tableau (23) with stress on the leftmost theme does not incur any violations of the *Infl-Stress constraints which are currently present in the ranking because the leftmost theme is not in the locality domain of the inflection.

(24)

**Table:** 

/ne=**p**-i-je-mo/	*Infl-Stress-1	*Infl-Stress-2	**Rightmost**
a. ˈne pijemo			***!
☞ b. neˈpijemo			**
c. ne piˈjemo		*!	*
d. ne pijeˈmo	*!	*	

Note that candidates a. and b. are not distinguished in terms of violations of the *Infl-Stress constraints. Similarly to Tableau (19), the correct result is due to the activity of **Rightmost** . We will return to this issue below and argue that ultimately, the rejection of stress by the negation particle has a different explanation, based on it being non-lexical material.

Next, we turn to the prefixed version of *piti∼pijemo*: *popiti∼popijemo.* The paradigm is shown in (25).

(25)

**Table:** 

a.	*ˈpop-i-ti*
	‘to drink up’
b.	*ˈpop-i-je-mo*
	‘we drink up’
c.	*ne ˈpopi-je-mo*
	‘we don’t drink up’

The infinitive form, which only has one theme vowel, can be handled using our current ranking, as shown in the tableau in (26).

(26)

**Table:** 

/**po-p**-i-ti/	*Infl-Stress-1	*Infl-Stress-2	**Rightmost**
☞ a. ˈpopiti			**
b. poˈpiti		*!	*
c. popiˈti	*!	*	

The present-tense form *ˈpop-i-je-mo* is the first one where our current ranking predicts the wrong output: *poˈp-i-je-mo. Note that, unlike in our prose above, in our OT analysis so far, there has been no distinction between theme vowels outside the locality domain of the inflection and genuine lexical material. In order to make this distinction we add the final constraint to the *Infl-Stress family:

**Table:** 

*Infl-Stress-3:	No stress on non-lexical morphemes (theme vowels, negation, inflection).

Including this last *Infl-Stress constraint in the ranking captures the present-tense forms of longer verbs with multiple theme vowels, as shown in the tableaux in (27) and (28).

(27)

**Table:** 

/**po-p**-i-je-mo/	*Infl-Stress-1	*Infl-Stress-2	*Infl-Stress-3	**Rightmost**
☞ a. ˈpopijemo				***
b. poˈpijemo			*!	**
c. popiˈjemo		*!	*	*
d. popijeˈmo	*!	*	*

(28)

**Table:** 

/ne=**po+p.**i-je-mo/	*Infl-Stress-1	*Infl-Stress-2	*Infl-Stress-3	**Rightmost**
a. ˈne popijemo			*!	****
☞ b. neˈpopijemo				***
c. ne poˈpijemo		*!	*	**
d. ne popiˈjemo		*!	*	*
e. ne popijeˈmo	*!	*	*	

Note that the inclusion of *Infl-Stress-3 in the ranking does not change anything for the already analyzed forms listed in (21), since all the vowels in these forms are part of non-lexical material and therefore all the candidates in (22), (23) and (24) violate *Infl-Stress-3. However, the inclusion of *Infl-Stress-3 slightly changes the analysis of negatives in tableaux (19) and (24). Previously, the non-stressing of the negation particle *ne* was accounted for by **Rightmost** ; now these forms are brought under the scope of *Infl-Stress-3.

Since *Infl-Stress-3 was the final constraint in the *Infl-Stress family, we can present the final overview of stressable morphological domains and *Infl-Stress violations that stressing them incurs. This overview is presented in Table 5.

**Table 5:** Overview of stressable morphological domains and penalizations by *Infl-Stress .

Stress on	Inflection	Theme vowels in the locality domain of inflection	Theme vowels outside of the domain of inflection, negation particle	Lexical material (roots, derivational affixes)
Penalized by	*Infl-Stress-1 *Infl-Stress-2 *Infl-Stress-3	*Infl-Stress-2 *Infl-Stress-3	*Infl-Stress-3	**/**

In even longer forms with multiple theme vowels, the stress will always end up rightmost on the lexical material, due to **Rightmost** , as illustrated by the present tense form *ispit-u-je-mo* ‘we test’ in (29).

(29)

**Table:** 

/**is+pit**-u-je-mo/	*Infl-Stress-1	*Infl-Stress-2	*Infl-Stress-3	**Rightmost**
a. ˈispitujemo				****!
☞ b. isˈpitujemo				***
c. ispiˈtujemo			*!	**
d. ispituˈjemo		*!	*	*
e. ispitujeˈmo	*!	*	*	

This concludes our analysis of nontransparent verbs. We have established the parsing algorithm for Serbian stress standard SC and completed the unpacking of the **StemStress** constraint into a family of constraints militating against stress on non-lexical material on three different levels: inflection (*infl-Stress-1), non-lexical material in the locality domain of inflection (*infl-Stress-2) and non-lexical material altogether (*infl-Stress-3).

The careful reader will have noticed that there still remains one form in Table 4 which is not captured by our ranking: the infinitive form *ˈškolov-a-ti*. Our ranking predicts *škoˈlov-a-ti. Once again, the predicted form is exactly what we encounter in Croatian stress standard varieties, indicating that this is indeed a plausible stress placement for this word in stress standard SC. However, the Serbian varieties allow much more prosodic contrast in this type. We turn to this last piece of the puzzle in the next section.

## 4 Denominal verbs

In this section we present an analysis of the type *ˈškolov-a-ti*, which we will argue to be denominal. In (30) we show the prosodic variation attested in this class, using the most common type of verbs: those which are related to an attested noun. In each case, we first present the related noun, whose stress pattern gets preserved in the verb.

(30)

**Table:** 

a.	*ˈškol-a*
	‘school’
	*ˈškolov-a-ti*
	‘to educate’
	*ˈškol-u-je-mo*
	‘we educate’
	*ne ˈškol-u-je-mo*
	‘we don’t educate’
b.	*ˈsavet*
	‘advice’
	*ˈsavetov-a-ti*
	‘to advise’
	*ˈsavet-u-je-mo*
	‘we advise’
	*ne ˈsavet-u-je-mo*
	‘we don’t advise’
c.	*trijumf*
	‘triumph’
	*ˈtrijumfov-a-ti*
	‘to triumph’
	*ˈtrijumf-u-je-mo*
	‘we triumph’
	*ne ˈtrijumf-u-je-mo*
	‘we don’t triumph’
d.	*koˈmand-a*
	‘command’
	*koˈmandov-a-ti*
	‘to command’
	*koˈmand-u-je-mo*
	‘we command’
	*ne koˈmand-u-je-mo*
	‘we don’t command’

Note that the hyphens in (30) separate lexical material, theme vowels and inflection. The parsing is made assuming the parsing algorithm from (20) above. However, this parsing is not crucial for the computation of the stress pattern in the type under consideration here, as it depends solely on the stress of the related noun.

As briefly discussed in Section 2, nouns have free stress placement in stress standard SC. This means that **NounFaith** (Smith 2001) is ranked above the constraints which enforce fixed stress in verbs, whereas general **Faith** is below the part of the ranking we have discussed so far. As a matter of fact, no further additions to the ranking are necessary in order to cater to the verbs of the *ˈškolov-a-ti*. What is necessary is the truly denominal representation along the lines of the tree in (31). Moreover, the nominal structure has to be visible to the phonological grammar, as it is in nouns.

(31)

**Table:** 

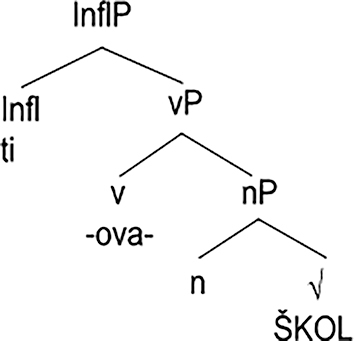

While the representation in (31) follows the conventions of Distributed Morphology (Halle and Marantz 1993), our model is compatible with any approach which allows verbs to incorporate nominal structure.

In (32) we show the tableau for *ˈškolov-a-ti.* We added **NounFaith** to the top of the ranking and general **Faith** to the bottom.

(32)

**Table:** 

/**[ˈškol]_nP_ov**-a-ti/	**NounFaith**	*Infl-Str-1	*Infl-Str-2	*Infl-Str-3	**R-most**	**Faith**
☞ a. ˈškolovati					***	
b. škoˈlovati	*!				**	*
c. školoˈvati	*!		*	*	*	*
d. školovaˈti	*!	*	*	*		*

If the same input without the visible nominal structure is submitted to our ranking, the winning candidate is the Croatian stress standard form *škoˈlov-a-ti.*

(33)

**Table:** 

/**ˈškolov**-a-ti/	**NounFaith**	*Infl-Str-1	*Infl-Str-2	*Infl-Str-3	**R-most**	**Faith**
a. ˈškolovati					***!	
☞ b. škoˈlovati					**	*
c. školoˈvati			*!	*	*	*
d. školovaˈti		*!	*	*		*

The tableau in (33) illustrates an important property of the stress standard system: no input stress marking on verbs will influence the surface form unless it comes under a nominal embedding.

This is another context where stress informs our morphosyntactic analysis. In Serbian stress standard verbs, all cases of exceptional stress are concentrated in the type *ˈškolov-a-ti∼ˈškol-u-je-mo.* Not surprisingly, this type is also used for loanword integration in present-day contact with English, as attested by several recent borrowings in our sample, such as *ˈlajkov-a-ti∼ˈlajk-u-je-mo* ‘like’ and *ˈstartov-a-ti∼ˈstart-u-je-mo* ‘start’. These verbs can be analyzed as denominal, since related nouns *lajk* and *start* are also attested. As a matter of fact, all verbs from current contact with English seem to be able to follow the denominal path.

An advantage of using **NounFaith** rather than special faithfulness that protects loanwords (see Simonović 2015 for an overview) lies in the fact that the former allows us to model the distribution of exceptional stress in a more parsimonious way. Recall that in Serbian stress standard SC only verbs of this type can have stress that is not stem-final. To use another comparison with the Croatian stress standard, the general SC noun *ˈvečer-a* ‘dinner’ is related to the verb *večer-a-ti ‘*to have dinner*’*. In Croatian stress standard varieties the stress pattern is preserved *ˈvečer-a-ti*, whereas in Serbian stress standard the only allowed stress pattern is *veˈčer-a-ti.* Our way of analyzing the impossibility of verbs like *ˈvečer-a-ti* in the Serbian stress standard would be that Serbian stress standard varieties only allow the incorporation of nominal structure in one type of verbs: those of the type *-ov-a-ti∼-u-je-mo*.

While a similar analysis is possible assuming **LoanFaith** as well, synchronically, it appears more parsimonious to assume that the verbalizing suffix *-ov-a-ti∼-u-je-mo* has a lexical entry that specifies it as combining with nominal structure. While this unification hypothesis needs to be tested against more data, if confirmed, it would lend support to the hotly debated view defended by Moravcsik (1975: 111–112) that “if verbs are borrowed, they seem to be borrowed as if they were nouns: the borrowing language employs its own means of denominal verbalization to turn the borrowed forms into verbs before using them as such”. The strongest version of this view would mean that denominal structure is imposed on all borrowed verbs, regardless of the status of the noun they contain.

Assuming the strongest version of the Moravcsikian analysis, we would also assign a denominal structure to borrowed verbs that do not properly contain a noun, e.g., *ˈkorigov-a-ti∼ˈkorig-u-je-mo* ‘correct’ (where *korig is not a word). Out of 169 verbs in *-ov-a-ti∼-u-je-mo* in our sample, 13 verbs can be analyzed as either containing a noun or being borrowed (*ˈlajkov-a-ti∼ˈlajk-u-je-mo*), 78 can be analyzed as properly containing a noun (*ˈškolov-a-ti∼ˈškol-u-je-mo*), and 78 as borrowed (*ˈkorigov-a-ti∼ˈkorig-u-je-mo*). The remaining 13 are native, yet not properly containing a noun. For instance, the verb *ˈnegodov-a-ti∼ˈnegod-u-je-mo* ‘object’ does not contain the noun *negoda. In such cases our analysis needs to postulate a noun that is not attested as a separate lexical entry (and all speakers share the intuition that it would be stressed as ˈ*negoda*), but only within the verbal structure. One promising possibility is offered by the fact that many of these 13 items sound as part of a relatively high register, so they may even be analyzed as borrowed from the pitch-accent varieties.

In sum, based on the fact that the type in *-ov-a-ti∼-u-je-mo* is densely populated by denominal items, but also hosts newly borrowed verbs from English, we argued that this type only contains denominal items and therefore constitutes an island of free stress among the verbal types in Serbian stress standard. Hence, nPs can be added to our overview of morphological domains to which our stress assignment is sensitive in Table 5. It would be the only domain where stress is not only not penalized, but where even input stress specifications are respected.

## 5 Conclusions

Starting from the typological generalizations that nouns allow more prosodic contrast than verbs and that stressed non-lexical material is avoided, we have developed an account of the interaction between the two. While making use of independently proposed mechanisms, our OT analysis is innovative in two important ways:

– In Section 3 we present arguments for replacing the constraint **StemStress** , militating against unstressed stems (a concept which may allow different implementations), with a family of more precisely defined constraints militating against stress on non-lexical material on three different levels: stress on inflection proper (*Infl-Stress-1), non-lexical material in the locality domain of inflection (*Infl-Stress-2) and non-lexical material altogether (*Infl-Stress-3).
– In Section 4 we presented an analysis of lexical-category effects not only between but also within categories. Denominal verbs are shown to allow the exceptional preservation of nominal stress, which we formalized as an effect of **NounFaith** .

Descriptively, we encountered a system that allows a variety of surface patterns. The only pattern excluded in all verbs is that with stress on agreement morphology (indicating that *Infl-Stress-1 is undominated). Despite the surface diversity of stress patterns, we showed that stress standard SC is a system in which verbs don’t have lexical prosody. An overwhelming majority of verbs have stress on the rightmost syllable of the lexical material. All cases where lexical material was not stressed on the rightmost syllable are restricted to a single conjugation and analyzable as denominal (and therefore under the auspices of **NounFaith** ). The few cases where inflection-adjacent theme vowels are stressed (in violation of *Infl-Stress-2) were found in verbs that had no stressable lexical material. Finally, the cases where the negation particle was stressed (in violation of *Infl-Stress-3) were cases where stressing the negation particle was the only way of avoiding a violation of *Infl-Stress-2 i.e., where there was no stressable lexical material **.**

Our approach to the analysis of morphologically nontransparent forms has been admittedly prosody-centric. Prosody was assumed to be a reliable indicator for morphosyntactic structure. It is our hope that future research will test our findings against further morphological and syntactic data.

## Data availability statement

The data underlying this analysis may be viewed in Simonović (2022). Stress placement in stress-standard Serbo-Croatian (speakers from Bor, Niš, Zaječar) [Data set]. https://doi.org/10.5281/zenodo.6606901.
